# Citizens’ Perceptions of Research and Innovation Dilemmas: Insights from a Large-Scale Survey in Four European Regions

**DOI:** 10.1038/s41597-023-02384-9

**Published:** 2023-07-20

**Authors:** Katharina Fellnhofer, Margarita Angelidou, Thomas Bakratsas, Chiara Buongiovanni, Toni Eiser, Elena Hörndler, Anastasia Panori, Rene Wintjes, Gabriella Quaranta

**Affiliations:** 1 Swiss Federal Institute of Technology Zürich, ETH Zürich, Zürich, Switzerland; 2 Department of Sociology, HARVARD UNIVERSITY, Cambridge, USA; 3 Research and Innovation Management GmbH, Neumarkt an der Ybbs, Austria; 4 Q-Plan International Advisors P.C., Thessaloniki, Greece; 5 Aristotle University of Thessaloniki, Thessaloniki, Greece; 6 White Research SRL, Saint-Gilles, Belgium; 7 APRE - Agenzia per la Promozione della Ricerca Italiana, Rome, Italy; 8 University of Maastricht, Maastricht, Netherlands; 9 EMSO - European Multidisciplinary Seafloor and Water Column Observatory, Rome, Italy; 10 APRE - Agenzia per la Promozione della Ricerca Europea (Agency for the Promotion of European Research), Rome, Italy

**Keywords:** Politics, Socioeconomic scenarios, Psychology and behaviour, Political economy of energy

## Abstract

This study presents a valuable dataset supporting regional research and innovation systems in four European regions: Vestland (Norway), Kriti (Greece), Galicia (Spain), and Overijssel (Netherlands). It focuses on understanding citizens’ perceptions of research and innovation dilemmas within these regions. The dataset comprises 14 questions aligned with the Responsible Research and Innovation framework, evaluating stakeholders’ techno-moral attitudes towards technological change and socio-economic outcomes. A survey conducted between April and July 2020 gathered responses from 7,729 individuals, ensuring broad age and gender representation. This dataset is highly valuable for regional policymaking and policymakers’ engagement strategies, enhancing equity and effectiveness in addressing grand societal challenges. Research outcomes reveal citizens’ aspirations for developmental trajectories prioritizing quality-of-life, renewable energy, and support for innovative SMEs in their regions. The study contributes to existing research by highlighting limited citizen trust and expectations of effective government actions in addressing societal challenges at the regional level.

## Abstract (summary)

**Table:** 

Measurements	Priority areas and future trajectories, trade-offs between innovation and rising societal challenges, trust, public engagement
Technology Types	Survey method
Factor Types	Sex and age
Sample Characteristics – Organism	Homo sapiens
Sample Characteristics – Environment	Covid-19
Sample Characteristics – Location	Vestland (Norway), Kriti (Greece), Galicia (Spain), and Overijssel (Netherlands)

## Background & Summary

Research and innovation involves social, ethical, and ecological dimensions with both economic growth and dilemmas. Research and innovation must be socially acceptable and morally correct^1,2^, which is behind the term Responsible Research and Innovation (RRI) introduced by the European Commission to align research and innovation with European values^2^. Moreover, RRI supports the UN Sustainable Development Goals (SDGs), which emphasize the need for new governance mechanisms and joint actions by a diverse set of stakeholders^3,4^. In this context, the RRI2SCALE project aimed to engage citizens from four regions (Kriti, Galicia, Overijssel, and Vestland) by surveying their views regarding potential future trajectories of their region, along with concerns and moral issues that might arise in relation to RRI.

The RRI concept remains in its infancy and lacks a comprehensive understanding or consensus^5,6^. Von Schomberg^7^ defines RRI as a transparent, interactive process by which a diverse set of stakeholders with different backgrounds become mutually responsive partners in a sustainable innovation process. The European Commission introduced the following definition in 2012: “Responsible Research and Innovation refers to the comprehensive approach of proceeding in research and innovation in ways that allow all stakeholders that are involved in the processes of research and innovation at an early stage (A) to obtain relevant knowledge on the consequences of the outcomes of their actions and on the range of options open to them and (B) to effectively evaluate both outcomes and options in terms of societal needs and moral values and (C) to use these considerations (under A and B) as functional requirements for design and development of new research, products and services”. Most importantly, public participation is an essential ingredient of RRI^8^.

Societal problems and solutions are intricately linked to specific geographic regions, emphasizing the importance of a bottom-up transformative innovation policy approach. In line with this perspective, Schwaag Serger, Soete & Stierna^9^ advocate for a trust-building, place-based approach to innovation policy. Their research highlights the significance of fostering local trust and engagement to effectively address societal challenges and promote sustainable innovation.

Trust is an underlying psychological condition in which we accept vulnerability because we expect positive intentions or behaviours from another^10^. Trust also acts as a social glue that keeps actors in a partnership (e.g.^11^,). Its multidimensional conceptualization highlights its key role in policymaking^10^. Trust also represents an alternative governance mechanism partly because it increases confidence in other parties’ commitment to a greater good^11^. Previous research has highlighted the innovative impact of trust at the individual level of analysis^12^ in such a way that trust in an organization predicted innovative behaviour and at the organizational level^13^ in such a way that a trust-filled climate positively affected personal initiative. Thus, citizen engagement has consistently been promoted in research and policymaking. In extension, citizens’ techno-moral attitudes are often influenced by their level of trust in the government’s commitment to implementing just, realistic, and sustainable policies. The presence or absence of this trust can significantly shape their perceptions and beliefs. For instance, their trust in the objectivity, inclusivity, and transparency of new policies may impact their techno-moral attitudes^14^. This highlights the interconnectedness between trust, government policies, and citizens’ perspectives, underscoring the need for building and maintaining trust to foster positive techno-moral attitudes in society. To our knowledge, no study has yet focused on the specific relationship between trust and citizen demands for engagement in regional policymaking, with particular attention to tackling grand societal challenges.

However, innovation can cause dilemmas; that is, situations requiring difficult decisions that entail undesirable outcomes for at least some stakeholders^15^. Dilemmas include trade-offs between the environment and economy^16^, and RRI seeks to overcome these trade-offs by foreseeing possible conflicts between perspectives^1,17^. Against this background, it is essential to design regional R&I policies that avoid or harmonize dilemmas at the regional level in an early stage of the innovation process^15^. This can be achieved by effectively engaging local stakeholders. Participatory smart innovation can overcome unattractive trade-offs, because innovation can satisfy diverse values simultaneously^18^.

The RRI2SCALE project aimed to address key aspects of transparency, participation, and information exchange among regional stakeholders, recognizing their crucial role in establishing and sustaining robust RRI governance structures. This manuscript presents a valuable dataset^19^ that contributes both methodologically and empirically to the mapping, analysis, and understanding of R&I dynamics within the four pilot regions. It specifically explores the future trajectories of R&I in the “smart cities - transport - energy” domain, under the framework of Responsible Research and Innovation (RRI). For a more comprehensive overview, a detailed summary of the project can be found on the project homepage (https://rri2scale.eu/index.php/resources/reports/), specifically in deliverable 1.4. This summary provides additional insights into the project’s objectives and outcomes.

## Methods

### Participants and data collection

Our methodological approach was supported by four regional survey companies (Palmos Analysis P.C., Newcom Research and Consultancy B.V., Newton Research Europe d.o.o., and Kantar A.S.) that used local languages to collect data between April and July 2020 through online surveys of a total of 7,729 individuals in Greece’s Kriti region (N = 2010), the Overijssel region of the Netherlands (N = 1660), Spain’s Galicia region (N = 2006), and Norway’s Vestland region (N = 2053). We did not exclude any subjects from our analysis. We deliberately recruited samples broadly representative as to age and gender (3,861 men; 3,830 women) in each region, with the following stakeholder distribution: 5.6% academics, 5.2% policymakers, 18.1% entrepreneurs, 51.5% in civil society, and 19.7% others. The data set consists of responses to 14 questions, of which five are demographic. The questionnaire was developed through a collaborative process involving the scientific committee of the project, comprising academic experts in the field of Responsible Research and Innovation (RRI). Valuable insights from these committee meetings formed the foundation for the design of the questionnaire. Detailed information regarding the key findings and insights from these meetings can be found in deliverable 1.1 of the project, accessible at: https://rri2scale.eu/wp-content/uploads/2022/01/D1.1-The-Regional-Dilemma.-report-on-how-EU-regions-integrate-RRI-in-territorial-RI-Landscape.pdf. It is important to emphasize that prior to participating in the survey, all respondents were required to provide electronic informed consent, ensuring their voluntary participation and protection of their rights. Where required, regional data collection was approved by relevant ethics committees (e.g., University of Twente BMS Ethical Committee, no. 200184). All data, analysis code, and research materials are available at 10.17605/OSF.IO/KPSBW^19^.

### Measurements

This section presents and articulates the individual survey questions into the 4 initial grouped sections: “priority areas/future trajectories”, “potential trade-offs”, “trust”, and “public engagement”. It also relates the sections with the six core dimensions of RRI, meaning: (i) ethics, (ii) public engagement, (iii) gender equality, (iv) science education, (v) open access, and (vi) governance.

Priority areas & future trajectories (related to ethics, public engagement, and gender equality): We identified priority areas and future trajectories in each region. This was a critical step to capture prerequisites for the RRI key of public engagement in innovation processes. The insights also contributed to underlying ethical trade-offs in each region as expressed by regional stakeholders. As such, regional authorities had access to a comprehensive list of priorities towards more ethically acceptable choices. On a scale of low, medium, and high priority, stakeholders had to indicate which areas they would choose to have their region spend money on, choosing from the following list: (1) provide grants for research and innovation activities, (2) provide support to small- and medium-sized businesses to develop and apply new technologies, (3) support citizens and companies to use renewable energy sources by subsidising them, (4) create new digital public services that are more easily accessed, (5) upgrade transport facilities (rail, road, or air) by making them smarter and more efficient, (6) skills development through vocational training activities, (7) create new public spaces and infrastructures that serve regional specific social or environmental needs, (8) or other. Regarding future trajectories, citizens were allowed to choose a maximum of two answers to the following question: In the long run, I believe that my region should be mainly shaped by (1) a hub of innovative small- and medium-sized businesses that will attract highly skilled workers, (2) a highly digitalised public sector region that uses citizen-friendly applications, (3) an energy-efficient region characterised by optimised energy production processes using renewable energy sources, (4) a high-mobility region that uses smart transport for optimising access, distances, and time, or (5) a high-quality-of-life region where citizens are heard and participate in addressing their daily life needs. By capturing sex-related trends (through demographic questions) in these questions, we provided policy makers with knowledge on how regional priority areas and future trajectories that are gender-sensitive.

Potential trade-offs in innovation vs. societal challenges (related to ethics): We asked stakeholders’ opinions of potential conflicting aspects between innovation and growing societal challenges, such as negative environmental effects or the exclusion of certain social groups from policy design processes. We used a five-point Likert scale from strongly disagree (1) to strongly agree (5) to quantify regional dilemmas through each individual’s levels of agreement with the following statements: (1) I believe that promoting innovation should be a higher priority than citizens’ well-being (such as jobs, income, housing, health, safety); (2) I believe that innovation should be boosted even though it might create gender inequalities in my region; (3) I believe that it is good to support innovation when it has a positive impact on smart cities, energy, and transport, even if it requires access to my personal data; (4) I believe that innovation outcomes for facilitating smart cities, energy, and transport should be boosted, even if I might not have all the skills needed to use them. This part of the measurements offered us a clearer understanding of how regional innovation processes can actively integrate ethics as key component of RRI, by anticipating and proactively addressing rising societal dilemmas.

Public general and institutional trust (related to open access and public engagement): Through this variable, we explored general and institutional trust related to innovation, as well as citizens’ willingness to participate in regional innovation policy design processes. We used a five-point Likert scale from strongly disagree (1) to strongly agree (5) to quantify trust indicators, using a composite measure that averaged each individual’s answer to the following four questions: in terms of general trust, I trust organizations or groups of people when they (1) assess the effects of innovation in an independent way (autonomy), (2) look at the effects of innovation from different angles (diversity), (3) clearly indicate which interests they have in innovation (interest), and (4) communicate in an open way about innovation (transparency). Regarding trust in regional organizations, we used a five-point Likert scale from not at all (1) to very much (5) to quantify trust indicators, using a composite measure that averaged each individual’s levels of agreement with eight statements gauging their trust in regional institutions: (1) regional government, (2) local government, (3) civil society organizations, (4) non-governmental organizations, (5) researchers, (6) small- and medium-sized businesses, (7) large companies, and (8) gender-balanced governing bodies. Essentially, the answers provided useful input on how to foster public engagement towards RRI. Moreover, the enhancement of institutional and general trust relates to open access principle of RRI, since regional institutions can function as bridges between local citizens and science (e.g., open share of scientific data that inform regional innovation decision making).

Stronger civic participation and public engagement (related to public engagement and governance): The next part of the “measurements” shed light on requirements of public engagement in regional science and innovation processes. We used a five-point Likert scale from strongly disagree (1) to strongly agree (5) to quantify demand for engagement in regional policymaking, using a composite measure that averaged each individual’s levels of agreement with two statements: (1) I believe that citizens should be actively involved in helping to design regional innovation policies, and (2) I believe that citizens should be actively involved in helping to evaluate regional innovation policies. Moreover, as another proxy question for public engagement, we asked participants how they would prefer to get involved in public dialogues. They had to choose two of the following options: (1) individual direct communication (e.g., individual meetings, letters), (2) online channels (e.g., platforms, social media accounts); (3) formal working groups (online and in-person) representing a community or group; (4) open events organized by public authorities (e.g., workshops); and (5) other. This was complemented by another question about “Experience with public dialogues”. Using a yes/no question, we asked if participants had ever been involved in public dialogues and then queried the extent (not at all, yearly, monthly, weekly, daily) to which they were willing to be involved in future public dialogues related to smart cities, transport, and energy. These questions targeted the RRI key of “governance”, as they help regional policy makers detect blind spots and civic preferences on how to build effective multi-stakeholder governance systems in regional innovation. Furthermore, this “measurements” part of the data descriptor is indirectly related to the dimensions of “science education”, as evidence-based policy investments in regional public dialogues are expected to enhance scientific literacy of participating citizens.

Demographics (related to gender equality): We asked about gender (female, male, prefer not to mention), age group (18–24, 25–34, 35–65, 66 or older), educational level (no or primary education, secondary education, higher education (bachelor’s degree or equivalent), higher education (master’s, PhD, or equivalent), activity status (employed, unemployed, retired, student, household activities, other), and kind of stakeholder (academia or research, government, business, civil society). The gender of participants served as a critical background variable that enabled the interpretation of all other questions through the gender lenses, supporting the overall implementation of gender equality measures in regional innovation policy making.

### Citizens participating as stakeholder

One particularly intriguing variable within the dataset is the inclusion of different stakeholders. It is uncommon to find comprehensive information on various stakeholders within a single dataset. Traditionally, research and innovation efforts have primarily focused on science and industry, with government later being recognized as a third stakeholder (triple helix). However, there is a growing acknowledgment of the relevance of civil society as a fourth stakeholder (quadruple helix). Schwaag Serger, Soete & Stierna^9^ highlight the challenge of defining and identifying these relevant actors but emphasize the importance of a trust-building, place-based approach to innovation policy that engages citizens, entrepreneurs, local communities, cities, and regions (p.15).

Incorporating data on such a diverse range of actors presents a unique challenge. In the survey, citizens were asked to identify their participation as part of academia or research, government, business, civil society, or other. The resulting distribution of stakeholders indicates that nearly 20% of participants identified themselves as “other,” suggesting the need for a more detailed characterization of stakeholder groups within society. This is particularly relevant for Galicia and Overijssel, where further exploration of stakeholder profiles would be beneficial. Overall, the distribution of the four provided stakeholder types is reasonably balanced. However, it is worth noting that in Kriti and Vestland, citizens demonstrated a higher inclination to participate as part of civil society, while their participation as part of the government was relatively lower.

These findings shed light on the diverse stakeholder landscape and highlight the importance of considering the preferences and perspectives of different stakeholder groups in regional innovation initiatives. Understanding these dynamics can inform targeted engagement strategies and facilitate more inclusive and effective innovation policies tailored to specific regions.

## Data Records

Questionnaire files and data records are available in XLSX format from the Open Science Framework (OSF) platform. The data sets have been anonymized to remove any personal information. Abbreviation guides for variable names are also included in each XLSX file. All data are merged in a single XLSX file with 11 tabs (see Table 1). All data for all four regions are consolidated on a single tab. There is also one tab outlining the code book used to transform the answers into numbers (see Table 2). We have also stored the metadata of each region and the original surveys in this XLSX file. We used OSF as the repository to host this information. The English version of the survey is provided as Supplementary Material; it was translated into the primary language of each region: Dutch in Overijssel, Greek in Kriti, Galician in Galicia, and Norwegian in Overi Vestland. Those surveys are provided as Word and PDF documents stored in a folder on the OSF platform.

**Table 1:** Descriptions of tabs used in the Excel file including all collected data.

Code Book	Coding key incl. comments during data cleaning
Consolidated	Consolidated data of all four regions
Questionnaires	Questionnaires in original languages of all four regions
NO Vestland	Norway Vestland Data
GR Kriti	Greece Kriti Data
ES Galicia	Spain Galicia Data
NL Overijssel	Netherlands Overijssel Data
NO Raw Data	Raw data received from the region Vestland
GR Raw Data	Raw data received from the region Kriti
ES Raw Data	Raw data received from the region Galicia
NL Raw Data	Raw data received from the region Overijssel

**Table 2:** Code book used for answers.

Question	Answer	code	Question	Answer	code
**1**	Low Priority	1	**9**	Annually	2
	Medium Priority	2		Monthly	3
	High Priority	3		Weekly	4
	No opinion/No answer	4		Daily	5
	na	4		Nothing	1
				No opinion/No answer	0
**1**	Yes	1		na	1
	No	0			
	I do not know	2	**10**	Male	0
				Female	1
**3 & 6**	Strongly disagree	1		Prefer not to mention	2
	In disagreement	2		Gender neutral	3
	Neither agree nor disagree	3			
	Agree	4	**11**	18–24 years	1
	Strongly Agree	5		25–34 years	2
	No opinion/No answer	0		35–44 years	3
	na	0		45–54 years	3
				55–64 years	3
**5**	Nothing	1		65–74 years	4
	Not much	2		Over 75 years	4
	Indifferent	3			
	A little	4	**13**	Employed	1
	A lot	5		Unemployed	2
	No opinion/No answer	0		Retired	3
	na	1		Student	4
				Domestic activities	5
**7**	No	0		Other	6
	Yes	1		na	0
**8**	No	0	**14**	University or R + D + I organisations (Professor or Researcher)	1
	Yes	1		Public Administration	2
	No opinion/No answer	2		Private Company	3
	na	2		Organisation representing Civil Society	4
				na	0

## Technical Validation

The following analyses support the technical quality of the data set as to data reliability and the technical rigour of our approach: (1) statistical analyses of data characteristics, and (2) general discussions of the procedures adopted used to ensure reliable and unbiased data production, such as sample tracking systems and third-party support.

### Characteristics of the data

A strength of these data is the ability to evaluate techno-moral attitudes regarding technological change and socio-economic outcomes in four European regions: Vestland (Norway), Kriti (Greece), Galicia (Spain), and Overijssel (Netherlands). These data can help policymakers shape interventions to strengthen trust in those regions.

This dataset holds the strength of incorporating survey data from diverse European cities, enabling a comprehensive understanding of citizens’ perspectives across various urban contexts. By capturing a range of demographic, cultural, and socioeconomic factors, the dataset provides a robust foundation for analyzing and drawing insights that can account for the potential variations among the cities surveyed.

### Descriptive results

The socio-demographic characteristics are shown in Table 3. Using one-way analysis of variance, Table 4 outlines differences by region, stakeholder type, age, and gender. There were significant differences across those categorisations, highlighting the distinctive characteristics of each region.

**Table 3:** Sample descriptive statistics.

	Spain: Galicia (1)	Greece: Kriti (2)	Norway: Vestland (3)	Netherlands: Overijssel (4)	Overall
	(N = 2006)	(N = 2010)	(N = 2053)	(N = 1660)	(N = 7729)
*Stakeholder*					
Academic community (1)	58 (2.9%)	100 (5.0%)	48 (2.3%)	227 (13.7%)	433 (5.6%)
Government (2)	220 (11.0%)	3 (0.1%)	17 (0.8%)	159 (9.6%)	399 (5.2%)
Business (3)	595 (29.7%)	216 (10.7%)	47 (2.3%)	541 (32.6%)	1399 (18.1%)
Civil society (4)	192 (9.6%)	1691 (84.1%)	1920 (93.5%)	175 (10.5%)	3978 (51.5%)
Other (5)	941 (46.9%)	0 (0%)	21 (1.0%)	558 (33.6%)	1520 (19.7%)
*Profession status activity*					
Employee (1)	1242 (61.9%)	963 (47.9%)	1268 (61.8%)	810 (48.8%)	4283 (55.4%)
Unemployed (2)	325 (16.2%)	251 (12.5%)	34 (1.7%)	79 (4.8%)	689 (8.9%)
Retired (3)	176 (8.8%)	600 (29.9%)	504 (24.5%)	356 (21.4%)	1636 (21.2%)
Pupil or Student (4)	116 (5.8%)	86 (4.3%)	107 (5.2%)	214 (12.9%)	523 (6.8%)
Household (5)	64 (3.2%)	106 (5.3%)	32 (1.6%)	81 (4.9%)	283 (3.7%)
Other (6)	83 (4.1%)	4 (0.2%)	108 (5.3%)	120 (7.2%)	315 (4.1%)
*Education*					
No or primary education (1)	44 (2.2%)	207 (10.3%)	91 (4.4%)	21 (1.3%)	363 (4.7%)
Secondary education (2)	628 (31.3%)	886 (44.1%)	228 (11.1%)	996 (60.0%)	2738 (35.4%)
Bachelor’s degree or equivalent (3)	998 (49.8%)	702 (34.9%)	679 (33.1%)	445 (26.8%)	2824 (36.5%)
Postgraduate, doctoral, or equivalent (4)	336 (16.7%)	215 (10.7%)	497 (24.2%)	166 (10.0%)	1214 (15.7%)
Other	0 (0%)	0 (0%)	558 (27.2%)	32 (1.9%)	590 (7.6%)
*Age*					
18–24 (1)	119 (5.9%)	101 (5.0%)	92 (4.5%)	293 (17.7%)	605 (7.8%)
25–34 (2)	450 (22.4%)	180 (9.0%)	274 (13.3%)	208 (12.5%)	1112 (14.4%)
35–65 (3)	1329 (66.3%)	1248 (62.1%)	1233 (60.1%)	762 (45.9%)	4572 (59.2%)
65 ≤ (4)	108 (5.4%)	481 (23.9%)	454 (22.1%)	397 (23.9%)	1440 (18.6%)
*Gender*					
Male (0)	1000 (49.9%)	971 (48.3%)	1026 (50.0%)	864 (52.0%)	3861 (50.0%)
Female (1)	1001 (49.9%)	1039 (51.7%)	1027 (50.0%)	763 (46.0%)	3830 (49.6%)
Other (2)	5 (0.2%)	0 (0%)	0 (0%)	33 (2.0%)	38 (0.5%)

Notes. In Vestland, the categories of higher general education (5) and higher vocational education (6) were also available; for the present study, they were merged into higher education (Bachelor’s degree or equivalent) (3).

**Table 4:** Differences between regions, stakeholders and gender.

	ANOVA			
	*Regions*	*Stakeholders*	*Age*	*Gender*
*Stakeholder*				
Academic community (1)	F (3,7728) = 77.46, p < 0.001	x	F (3,7725) = 156.25, p < 0.001	F (3,7725) = 18.34, p < 0.001
Government (2)				
Business (3)				
Civil society (4)				
Other (5)				
*Profession status activity*				
Employee (1)	F (3,7728) = 56.10, p < 0.001	F (5,7723) = 107.42, p < 0.001	F (3,7725) = 524.01, p < 0.001	F (3,7725) = 22.56, p < 0.001
Unemployed (2)				
Retired (3)				
Pupil or Student (4)				
Household (5)				
Other (6)				
*Education*				
No or primary education (1)	F (3,7728) = 807.17 p < 0.001	F (5,7723) = 68.69, p < 0.001	F (3,7725) = 47.83, p < 0.001	F (3,7725) = 13.95, p < 0.001
Secondary education (2)				
Bachelor’s degree or equivalent (3)				
Postgraduate, doctoral, or equivalent (4)				
Other				
*Age*				
18–24 (1)	F (3,7728) = 91.34, p < 0.001	F (5,7723) = 133.51, p < 0.001	x	F (3,7725) = 58.89, p < 0.001
25–34 (2)				
35–65 (3)				
65 ≤ (4)				
*Gender*				
Male (0)	F (3,7728) = 0.42, p = 0.74, n.s.	F (5,7723) = 28.91, p < 0.001	F (3,7725) = 40.59, p < 0.001	x
Female (1)				
Other (2)				
*Region*				
Spain: Galicia (1)	x	F (5,7723) = 61.30, p < 0.001	F (3,7725) = 131.95, p < 0.001	F (3,7725) = 16.27, p < 0.001
Greece: Kriti (2)				
Norway: Vestland (3)				
Netherlands: Overijssel (4)				

## Supplementary information

Supplementary Information: Survey

## Data Availability

All data, analysis code, and research materials are available at 10.17605/OSF.IO/KPSBW^19^. Data has been deposited at 10.17605/OSF.IO/KPSBW^19^.
