# Supplementary material for: Citizens’ Perceptions of Research and Innovation Dilemmas: Insights from a Large-Scale Survey in Four European Regions
Source: Sci Data. 2023 Jul 20;10:473. doi: 10.1038/s41597-023-02384-9 (PMC10359344; doi:10.1038/s41597-023-02384-9)
Supplement: Supplementary file 1 — Supplementary Information: Survey [file 41597_2023_2384_MOESM1_ESM.docx]

### Supplementary Information: Survey

### SECTION 1: Identification of priority areas and future trajectories

In this section we would like to get your views regarding the most important areas for investing in your region. This will help us provide a comprehensive list of priorities to regional authorities based on your preferences. The second question aims at identifying insights about your perceptions regarding future trajectories of your region.

1. In the following list, please indicate which areas you would choose your region to spend money on.

|  | Low Priority | Medium Priority | High Priority |
| --- | --- | --- | --- |
| Provide grants for research and innovation activities |  |  |  |
| Provide support to small and medium sized businesses to develop and apply new technologies |  |  |  |
| Support citizens and companies to use renewable energy sources by subsidising them |  |  |  |
| Create new digital public services that are more easily accessed |  |  |  |
| Upgrade transport facilities making them smarter and more efficient (rail, road, or airports) |  |  |  |
| Skills’ development through vocational training activities |  |  |  |
| Create new public spaces and infrastructures that serve regional specific social or environmental needs |  |  |  |
| Other (Please specify: _______) |  |  |  |

1. In the long-run, I believe that my region should be mainly shaped as (max two options):
   - a hub of innovative small and medium sized businesses that will attract highly skilled workers
   - a highly-digitalised public sector region that uses citizen-friendly applications
   - an energy-efficient region characterised by optimised energy production processes using renewable energy sources
   - a high-mobility region that uses smart transport for optimising access, distances and time
   - a high quality of life region where citizens are heard and participate in addressing their daily life needs

**SECTION 2: Potential trade-offs between innovation and rising societal challenges**

In this section we ask your opinion about potential conflicting aspects between innovation and rising societal challenges, such as negative environmental effects or exclusion of specific social groups from policy design processes.

1. Please indicate your agreement with the following arguments:

|  | Strongly disagree | Disagree | Neither agree nor disagree | Agree | Strongly agree |
| --- | --- | --- | --- | --- | --- |
| I believe that promoting innovation should be a higher priority than citizens’ well-being (such as jobs, income, housing, health, safety). |  |  |  |  |  |
| I believe that innovation should be boosted even though it might create gender inequalities in my region. |  |  |  |  |  |
| I believe that it is good to support innovation when it has positive impact on smart cities, energy, and transport, even if it requires access to my personal data. |  |  |  |  |  |
| I believe that innovation outcomes for facilitating smart cities, energy and transport should be boosted, even if I might not have all the necessary skills to use them. |  |  |  |  |  |

**SECTION 3: Trust to local institutions and public participation**

In this section we want to explore general and institutional trust related innovation, as well as your willingness to participate in regional innovation policy design processes.

1. In terms of general trust, I trust organizations or groups of people when they:

|  | Strongly disagree | Disagree | Neither agree nor disagree | Agree | Strongly agree |
| --- | --- | --- | --- | --- | --- |
| assess the effects of innovation in an independent way |  |  |  |  |  |
| look at the effects of innovation from different angles |  |  |  |  |  |
| clearly indicate which interests they have in innovation |  |  |  |  |  |
| communicate in an open way about innovation |  |  |  |  |  |

1. Please indicate to what extent you trust the following organisations in your region.

|  | Not at all | Not much | Indifferent | A little | Very much |
| --- | --- | --- | --- | --- | --- |
| Regional government |  |  |  |  |  |
| Local government |  |  |  |  |  |
| Civil society organisations |  |  |  |  |  |
| Non-Governmental Organisations |  |  |  |  |  |
| Researchers/scientists working for universities |  |  |  |  |  |
| Small and medium businesses |  |  |  |  |  |
| Large companies |  |  |  |  |  |
| Gender balanced governing bodies |  |  |  |  |  |

1. Please indicate your agreement with the following arguments:

|  | Strongly disagree | Disagree | Neither agree nor disagree | Agree | Strongly agree |
| --- | --- | --- | --- | --- | --- |
| I believe that citizens should be actively involved in helping to design regional innovation policies |  |  |  |  |  |
| I believe that citizens should be actively involved in helping to evaluate regional innovation policies |  |  |  |  |  |

1. Which of the following ways would you choose if you wanted to get involved in public dialogues? (max 2)
   - individual direct communication (e.g. individual meetings, letters etc.)
   - online channels (e.g. platforms, social media accounts etc.)
   - formal working groups (online and live) representing a community or group
   - open events organized by public authorities (e.g. workshops)
   - Other (Please specify: _______)
2. Have you ever been involved in public dialogues?
   - Yes
   - No
3. To what extend are you willing to be involved in future public dialogues related to:

|  | Not at all | Yearly | Monthly | Weekly | Daily |
| --- | --- | --- | --- | --- | --- |
| smart cities |  |  |  |  |  |
| transport |  |  |  |  |  |
| energy |  |  |  |  |  |

### SECTION 4: Background information

1. Gender:
   - Female
   - Male
   - Prefer not to mention
2. Age group:
   - 18 – 24 years old
   - 25 – 34 years old
   - 35 – 65 years old
   - More than 65 years old
3. Please indicate your educational level:
   - No or Primary education
   - Secondary education
   - Higher education (Bachelor’s degree or equivalent)
   - Higher education (Master’s, PhD or equivalent)
4. Please indicate your activity status:
   - Employed
   - Unemployed
   - Retired
   - Student
   - Household activities
   - Other
5. You participate in this survey as part of:
   - Academia or research
   - Government
   - Business
   - Civil Society
6. Do you have any additional comments you would like to include? (optional)

(Free text)
